# The “Auto-Dissemination” Approach: A Novel Concept to Fight *Aedes albopictus* in Urban Areas

**DOI:** 10.1371/journal.pntd.0001793

**Published:** 2012-08-28

**Authors:** Beniamino Caputo, Annamaria Ienco, Daniela Cianci, Marco Pombi, Vincenzo Petrarca, Alberto Baseggio, Gregor J. Devine, Alessandra della Torre

**Affiliations:** 1 Dipartimento di Sanità Pubblica e Malattie Infettive, Università di Roma “Sapienza”, Rome, Italy; 2 Faculty of Veterinary Medicine, Utrecht University, Utrecht, The Netherlands; 3 Dipartimento di Biologia e Biotecnologie “Charles Darwin”, Università di Roma “Sapienza”, Rome, Italy; 4 I.N.D.I.A. INDUSTRIE CHIMICHE S.p.A, Padua, Italy; 5 Cairns Public Health Unit, Tropical Regional Services, Queensland Health, Cairns, Australia; Liverpool School of Tropical Medicine, United Kingdom

## Abstract

**Background:**

The main constraint to the fight against container-breeding mosquito vectors of human arboviruses is the difficulty in targeting the multiplicity of larval sources, mostly represented by small man-made water containers. The aim of this work is to assess the feasibility of the “auto-dissemination” approach, already tested for *Aedes aegypti*, as a possible alternative to traditional, inefficient control tools, against *Ae. albopictus* in urban areas. The approach is based on the possibility that wild adult females, exposed to artificial resting sites contaminated with pyriproxyfen, can disseminate this juvenile hormone analogue to larval habitats, thus interfering with adult emergence.

**Methodology:**

We carried out four field experiments in two areas of Rome that are typically highly infested with *Ae. albopictus*, i.e. the main cemetery and a small green area within a highly urbanised neighbourhood. In each area we used 10 pyriproxyfen “dissemination” stations, 10 “sentinel” sites and 10 covered, control sites. The sentinel and control sites each contained 25 *Ae. albopictus* larvae. These were monitored for development and adult emergence.

**Principal Findings:**

When a 5% pyriproxyfen powder was used to contaminate the dissemination sites, we observed significantly higher mortality at the pupal stage in the sentinel sites (50–70%) than in the controls (<2%), showing that pyriproxyfen was transferred by mosquitoes into sentinel sites and that it had a lethal effect.

**Conclusions:**

The results support the potential feasibility of the auto-dissemination approach to control *Ae. albopictus* in urban areas. Further studies will be carried out to optimize the method and provide an effective tool to reduce the biting nuisance caused by this aggressive species and the transmission risk of diseases such as Dengue and Chikungunya. These arboviruses pose an increasing threat in Europe as *Ae. albopictus* expands its range.

## Author Summary

*Aedes albopictus* (the Asian Tiger mosquito) is one of the most invasive and aggressive disease vectors in the world. It is a serious public nuisance and a public health risk, due to its ability to transmit pathogens to humans. The control of this mosquito is complicated by the difficulty in targeting either juveniles (due to their ability to colonise myriad and often cryptic domestic and natural water containers) or adults (due to their diurnal and outdoor resting and biting patterns). The aim of our work was to assess the feasibility of a novel approach to control *Ae. albopictus* in urban areas. This was based on the possibility that wild adult females, exposed to artificial resting sites contaminated with an insect growth regulator, could subsequently contaminate their breeding sites and kill both the larvae and pupae developing therein. The results obtained from field experiments carried out in highly infested areas in Rome demonstrate that this approach has potential as a valid alternative to traditional (and ineffective) larval control efforts in urban areas. We therefore introduce a new perspective in the fight against the Asian Tiger Mosquito.

## Introduction

*Aedes albopictus* (Skuse, 1894) is native to Southeast Asia. In recent decades this mosquito has invaded and efficiently colonized temperate areas of the US and Europe, thanks to passive transportation of eggs in used tires and the ability to produce diapausing eggs [1], [2]. In its tropical range the species is a secondary vector of human arboviruses, such as Dengue, while in temperate regions its impact on public health is mostly due to its aggressive and diurnal biting behavior. However, in recent years, *Ae. albopictus* has been the sole vector of large epidemics of Chikungunya virus in La Reunion, France [3], [4] and Kerala, India [5]. In August 2007 the species was responsible for a Chikungunya virus outbreak in the Province of Ravenna in north-eastern Italy where more than 250 human cases were confirmed [6] and where the mosquito has repeatedly been identified as infected by Chikungunya [7], [6], [8] and Usutu viruses [9]. In 2010 *Ae. albopictus* was responsible for 2 and 31 cases of endemic transmission of Dengue in France and China, [10], [11]. The report of a human blood index ≥80% in Rome raises particular concern for the potential of this species to vector pathogens in urban areas where humans represent the major blood-meal source [12].

In Italy, *Aedes albopictus* was detected for the first time in Genoa in 1990 and the next year in Padua [13], [14]. In the following years it spread to 19 out of 20 Italian regions and over 82 out of 107 Italian provinces [15]. It was first detected in Rome in 1997 [16] and since then it has colonized the whole urban area through three phases “a first massive spread, a following maintenance of infestation, and the colonization of alternative winter breeding sites with favourable climatic conditions” [17]. The successful invasion of Rome and of other urban areas in Italy and worldwide is driven by the ability of this mosquito to exploit a large variety of water containers as larval breeding sites, including the catch basins of storm drains, used tires, domestic containers, vases, etc. The difficulty in identifying and treating all these sources makes control extremely difficult. A survey carried out in Rome's zoological gardens, which estimated the larval density of mosquitoes breeding in catch basins, showed that a large number of productive alternative larval biotopes exist [18]. Despite this, the most commonly utilized strategy to reduce *Ae. albopictus* densities in Italy is the treatment of catch basins with larvicidal compounds [19]. This only targets an unknown percentage of the overall aquatic habitat. Indoor insecticidal treatments and house screening are not suitable due to the largely exophilic behavior of the species, and outdoor adulticidal treatments are recommended only in case of emergencies (e.g. virus outbreaks or very high nuisance in sensible locations such as outdoor recreational areas) due to their environmental impact and their low cost-efficiency ratio [19].

The aim of this work is to assess the feasibility of a new approach for the control of *Ae. albopictus* in urban areas, inspired by results obtained on the tropical Dengue vector, *Ae. aegypti*, in Thailand [20] and in Peru [21]. This approach, named “auto-dissemination”, is based on the possibility that wild adult mosquitoes exposed to artificial resting sites contaminated with pyriproxyfen (PPF, a juvenile hormone analogue), can disseminate insecticide to larval breeding sites, thus preventing adult emergence. This strategy is facilitated by the oviposition behaviour of both *Ae. aegypti* and *Ae. albopictus*, that typically scatter the eggs from a single gonothrophic cycle among several temporary sites. This increases the probability of at least some larvae reaching the adult stage [22]. Second, extraordinarily low doses of PPF (*Ae. aegypti*: LC_50_ = 0.011 p.p.b., [23], 0.0039 p.p.b. [24]; *Ae. albopictus*: LC_50_ = 0.11 p.p.b. [25], are needed to interfere with the metamorphosis of juvenile stages [26], and/or to cause morphological and functional aberrations in emerging adults, such as decreased fertility in males and females [27], [28]. Third, evidence from laboratory experiments shows that females either forced to walk on PPF-treated paper or topically contaminated can contaminate larval sites and significantly inhibit adult emergence [29], [30].

Thus, the “auto-dissemination” approach can be proposed as a ‘pull’ (i.e attraction of wild mosquitoes to PPF-contaminated sites for contamination) and ‘push’ (i.e. dispersal of contaminated mosquitoes and dissemination of PPF to larval habitats) control strategy with the potential to target the myriad of cryptic larval breeding sites that cannot be reached by traditional larvicidal applications.

We here present the results of four “auto-dissemination” experiments carried out in two areas of Rome with high infestations of *Ae. albopictus*: a cemetery and an enclosed garden.

## Materials and Methods

### Dissemination stations

**Figure 1:**
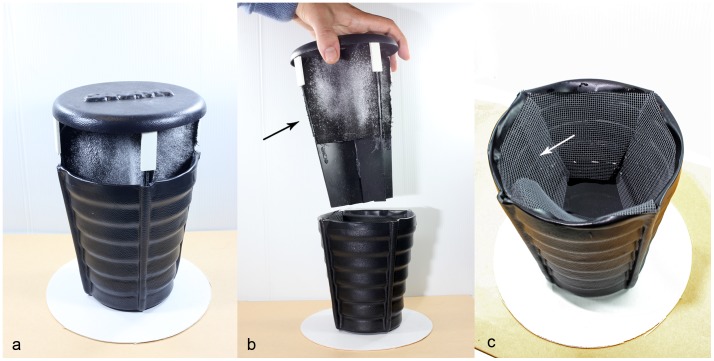
Dissemination station (DS) used for “auto-dissemination” experiments in Rome. a – whole DS; b – higher and lower parts of DS, where arrow indicates black cotton cloth dusted with powdered pyriproxyfen; c – lower part of DS, where arrow indicates the net placed above the water level to prevent mosquitoes contacting with water.

Dissemination stations (DS, Figure 1) were adapted from modified sticky traps (ST), previously developed by our group [31]. The four sticky surfaces were replaced by four 12×8 cm black cotton cloths (Figure 1b). A thick net was placed over the water to prevent mosquitoes from ovipositing (Figure 1c). Before each experiment, each DS was filled with 700 ml of tap water and each cloth was dusted with 1 g of powdered PPF. This was obtained by manually grinding 0.5% or 5% PPF tablets (Proxilar, I.N.D.I.A. Industrie Chimiche S.p.A.) to an granule average size of 40–80 micron.

### Sentinel sites and control sites

Larval/pupal mortality in sentinel sites (SS) that were potentially contaminated with PPF by wild mosquitoes was compared to mortality in uncontaminated control sites (CS). Sentinel sites were assembled by inserting a 600 ml Pyrex beaker into a standard ovitrap (i.e. a black vase). This was done to allow easy decontamination after the experiments (PPF adheres to plastics, but not to glass). The border of the beaker was covered with black tape to avoid reflections. Control sites were similar to SS but closed with white nets to prevent mosquitoes from entering the beakers and transferring PPF. Each SS and CS contained 200 ml of tap water, 0.07 g of cat biscuits and 25 third-instar *Ae albopictus* larvae. These were obtained from eggs collected from the “Sapienza” University campus by ovitraps during the weeks before the experiments. After hatching, the larvae were reared outdoors on the terrace of the Department of Public Health and Infectious Diseases of “Sapienza” University at a density of about 1 larva/ml.

### Study areas

The experiments were carried out in the following two sites located in a highly urbanized area of Rome (Italy) adjacentto the University “Sapienza” campus.

#### Site 1- Verano Cemetery

**Figure 2:**
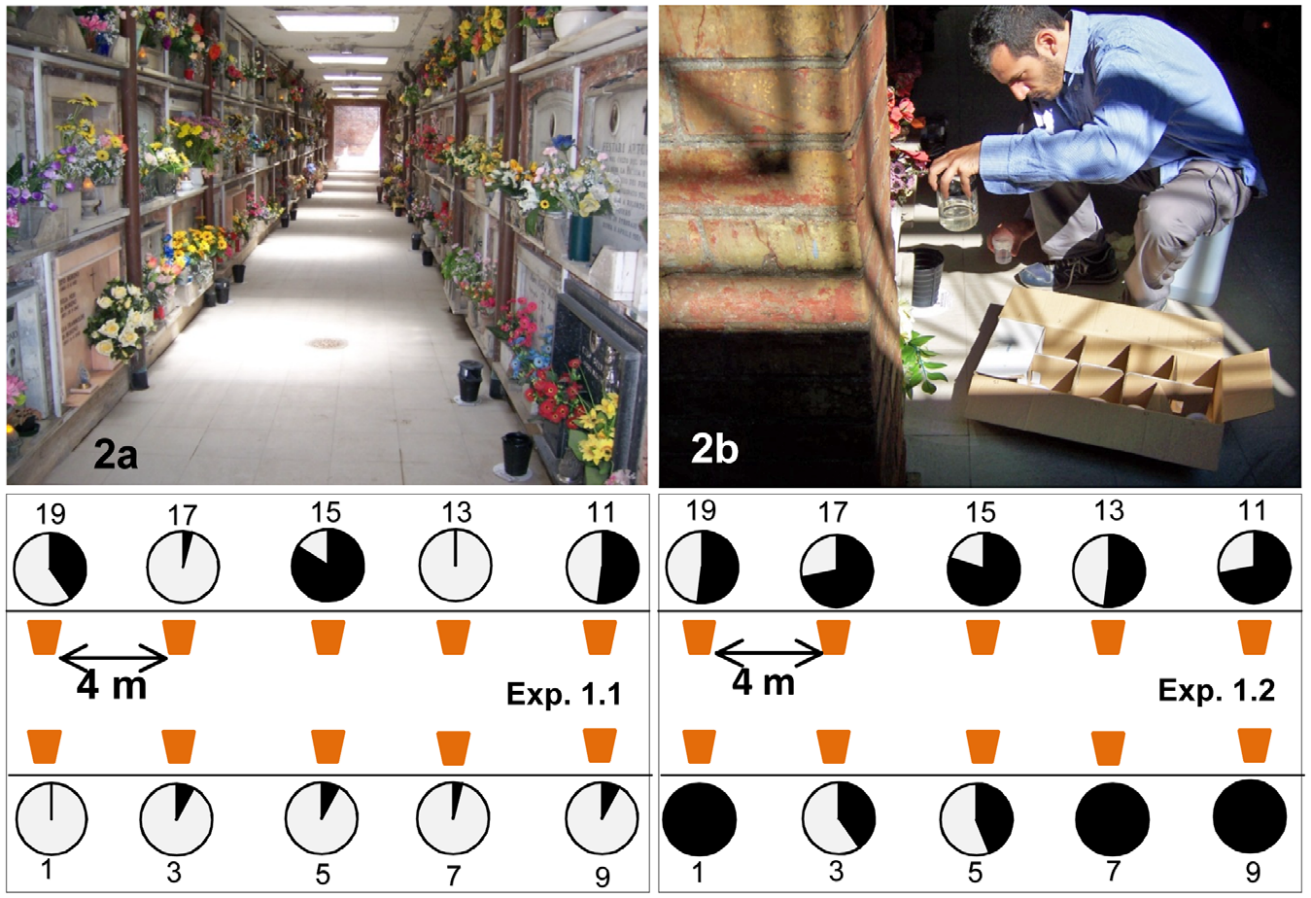
Ecology and results of “auto-dissemination” experiments in the Verano Cemetery in Rome (Italy). a - internal view of the burial corridor with dissemination stations and sentinel sites; b – monitoring of *Aedes albopictus* larval/pupal mortality; Exp. 1.1 and Exp. 1.2: frequencies (%) of dead larvae/pupae (in black) and of emerged adults (in white) in the first and second experiment carried out in the study area with 0.5% and 5% pyriproxyfen powder, respectively; sentinel sites are numbered accordingly to Figure 4.

Verano is the largest (98 ha) and the oldest cemetery in Rome that is still in use. It is a green area within an urban neighbourhood less than 500 m away from “Sapienza” University campus. Very high densities of *Ae. albopictus* have been reported, as is common in cemeteries due to the high number of potential larval breeding sites represented by flower vases [12]. The site chosen for the experiments (41°54′15.43″ N and 12°31′34.89″) is a shallow underground crypt formed by eight 22×2 m corridors with walls of tombs on both sides. Ten DS and 10 SS were placed in a single corridor, in pairs, as shown in Figures 2a. These were about 90 cm distant from each other and 4 m from the nearest other pair. Similarly, ten DS and 10 CS were placed in a second corridor located 50 m from the first one. Experiments were carried out from 17 to 24 August 2010, with 0.5% PPF powder (Exp. 1.1) and from 10 to 22 July 2011, with 5% powder (Exp. 1.2).

#### Site 2 – Enclosed garden

**Figure 3:**
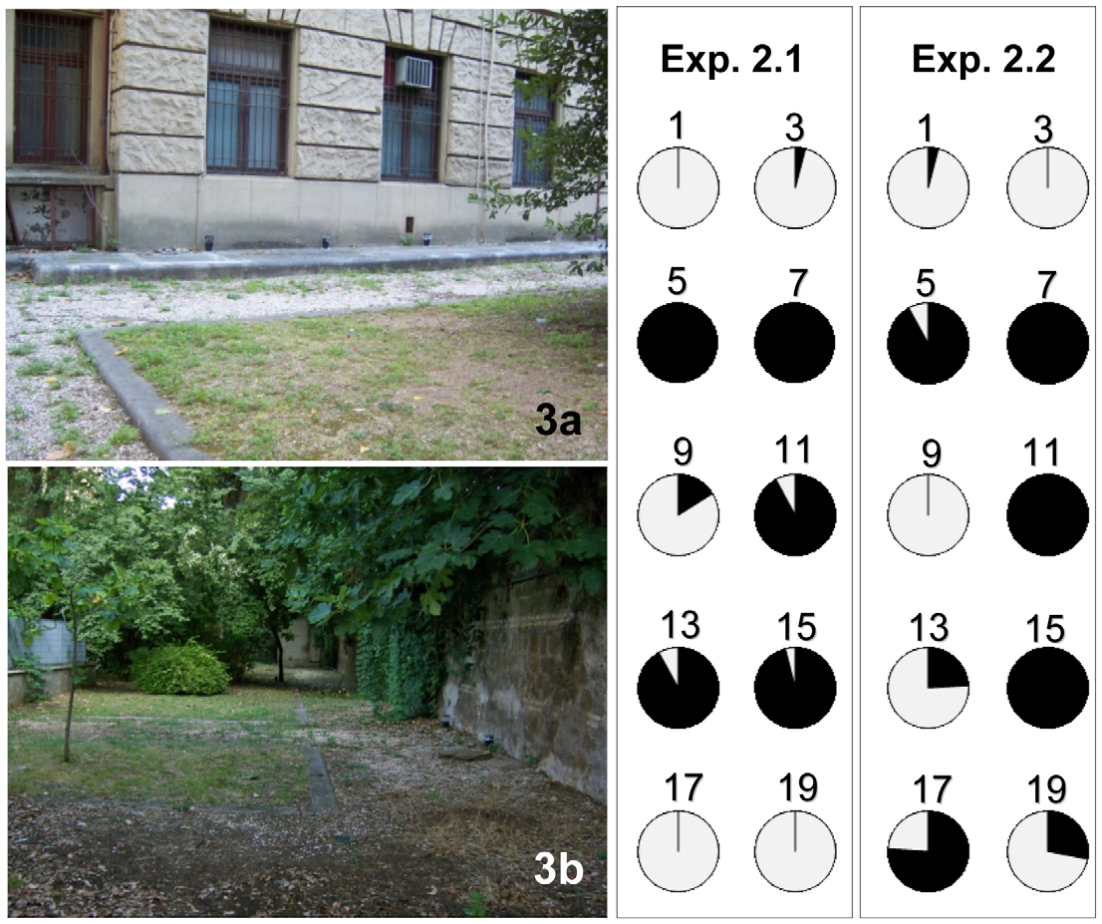
Ecology and results of “auto-dissemination” experiments in an enclosed garden in Rome (Italy). a and b – views of the study area with dissemination stations and sentinel and control sites; Exp. 2.1 and Exp. 2.2: frequencies (%) of dead *Aedes albopictus* larvae/pupae (in black) and of emerged adults (in white) in the first and second experiment carried out in the study area with 5% pyriproxyfen powder; sentinel sites are numbered accordingly to Figure 4.

The garden is part of the Institute of Anatomy and is less than 300 m from the main “Sapienza” University campus and about 850 m from the Verano cemetery (41°54′23.32″N and 12°30′57.35″E). The experiments were carried out in the 1 ha garden (Figures 3a and 3b), where 10 DSs dusted with 5% pyriproxyfen were placed at a distance of about 2 m right from an equal number of SS; 10 CS were also placed about 2 m from SS. Two replicates were carried out from 28 July to 11 August 2011 (Exp. 2.1) and from 26 August to 15 September 2011 (Exp. 2.2), respectively.

### Experimental procedures

During the experiments, SS and CS were monitored every two days. For each larval cohort, we derived cumulative totals of dead larvae, dead pupae and emerged adults, as follows: i) live larvae were left to develop further; ii) dead larvae and pupae were counted and removed (Figure 2b); iii) live pupae were counted and transferred by pipette to a separate disposable cup containing uncontaminated water; these cups were covered with netting and maintained under semi-natural conditions until adult emergence or pupal death; iv) the water level in the SS and CS was maintained at 200 ml volume; v) temperature was recorded using one data logger for each corridor in Site 1, and by individual data loggers located close to each SS in Site 2. Monitoring continued until all the original 25 larvae were either dead or emerged (7–12 days).

### Statistical analysis

Mortality in SS and CS was analysed by nonparametric alternatives to the t-test, since the distribution was not normal. We used the Kolmogorov-Smirnov (K-S) two-sample test (two-tailed), which is powerful even when the distributions differ in terms of dispersion We used Wilcoxon signed-rank tests (two-tailed) to compare the mortality in SS between experiments within same field site: this is a non-parametric paired difference test that compares two related samples or repeated measures on a single sample.

Two mixed-effect logistic regression models (one per study area) were applied to quantify PPF-related mortality. Each model includes, as independent variables, the number of replicates (“replicate effect”), the treatment (i.e. potential contamination in SS by wild mosquitoes *vs.* no contamination in CS) and the interaction between the replicates and the treatments, which allows an evaluation of the effect on the mortality in the sentinel sites in each replicate. The code identifying each SS and each CS was included as random effect to correct for the repeated measures within sites (“site-effect”).

## Results

Table 1 shows the overall mortality observed in SS and CS in the four experiments. Overall mortality in SS was ≥50% in the 3 experiments carried out with 5% PPF concentration, and 20.8% in the first experiment in Site 1, with 0.5% concentration. In all experiments, >90% of the mortality recorded in SS occurred at the pupal stage. In Site 2 however, about 13% of deaths occurred during adult emergence. This has been frequently noted in insects treated with PPF [32], but never in mosquitoes. The overall mortality in CS was ≤2.5%.

**Table 1:** *Aedes albopictus* mortality in control and sentinel sites during the experiments carried out in Rome.

		Mortality in CSs			Mortality in SSs		
PPF	T	Larvae	Pupae	Total	Larvae	Pupae	Total
0.5%	26.8°C	3	3	2.4%	3	49	20.8%
5%	23.8°C	0	3	1.2%	4	174	71.2%
5%	25.5°C	0	3	1.2%	1	124	50%
5%	25.8°C	1	3	1.6%	12	119	52.4%

PPF = pyriproxyfen concentration; T = mean daily temperature during experiments; Total = percentage of dead larvae/pupae over 250 third instar larvae in 10 control sites (CS) and 10 sentinel (SS), respectively.

In Site 1, a single experiment (1.1) utilised the 0.5% PPF formulation and a subsequent experiment utilised the 5% formulation (1.2). No significant differences in mortality in SS *vs.* CS were observed using the 0.5% concentration (K-S, p = 0.164; Figure 2). However, at 5% concentration mortality in SS was higher than that observed in CS (K-S, p<<0.001; Figure 2). Mortality in SS was lower in Exp.1.1 (median = 2, Q_1_ = 1, Q_3_ = 8) than in Exp 1.2 (median = 18, Q_1_ = 13, Q_3_ = 23.75) (significant difference shown by Wilcoxon test, p = 0.004).

In Site 2, both experiments (2.1 and 2.2) utilised the 5% concentration. The difference in mortality in SS *vs.* CS was significant only in Exp. 2.2 (K-S, p = 0.015; Figure 3), although the trend was confirmed in Exp. 2.1 (K-S, p = 0.055; Figure 3). No difference in mortality in SS was observed between the two replicates (Wilcoxon test, n.s.; Exp 2.1: median = 13.5, Q_1 = _0.25, Q_3_ = 23.75; Exp. 2.2: median = 13, Q_1_ = 2.25, Q_3_ = 24.5).

The results of the mixed-effect logistic regression model showed that mortality was always significantly higher in SS than in CS. In Site 1, mortality was 9- and 66.5-fold higher in SS than in CS- in Exp.1.1 and in Exp 1.2, respectively, although mortality observed in SS was approximately 3 times lower in Exp 1.1 than in Exp 1.2 (Table 2). In Site 2, mortality was 49- and 37-fold higher in SS than in CS in Exp.2.1 and Exp 2.2, respectively (Table 3). Mortality was similar in the two replicates and no significant differences were found in the interaction between “replicate” and “treatment”.

**Table 2:** Results of mixed effect logistic regression analysis on data obtained in Verano Cemetery, Rome.

VARIABLES	OR	95% CI	
Intercept	**0.004**	0.002	0.009
Exp 1.1 - SS *vs* CS	**8.942**	3.568	22.413
Exp 1.2 - SS *vs* CS	**66.500**	15.501	285.283
Treatment - Exp 1.2 *vs* Exp 1.1	3.179	0.741	13.637
No treatment - Exp 1.2 *vs* Exp 1.1	0.427	0.103	1.770

The model takes into account the effect of: the treatment (i.e, sentinel, SS, *versus* control, CS, sites), the replicates (i.e. Exp 1.1 and Exp 1.2) and the interaction between treatment and replicates on pyriproxyfen-related mortality. OR = odds ratio; CI = confidence interval. Statistically significant (p<0.05) odds ratios in bold.

**Table 3:** Results of mixed effect logistic regression analysis on data obtained in enclosed garden, Rome.

VARIABLES	OR	95% CI	
Intercept	**0.001**	0.000	0.004
Exp 2.1 - SS vc CS	**48.668**	8.181	289.515
Exp 2.2 - SS vc CS	**37.248**	7.177	193.304
Treatment - Exp 2.2 vs Exp 2.1	0.742	0.143	3.851
No treatment - Exp 2.2 vs Exp 2.1	0.97	0.191	4.932

The model takes into account the effect of: the treatment (i.e, sentinel, SS, *versus* control, CS, sites), the replicates (i.e. Exp 2.1 and Exp 2.2) and the interaction between treatment and replicates on pyriproxyfen-related mortality. OR = odds ratio; CI = confidence interval. Statistically significant (p<0.05) odds ratios in bold.

**Figure 4:**
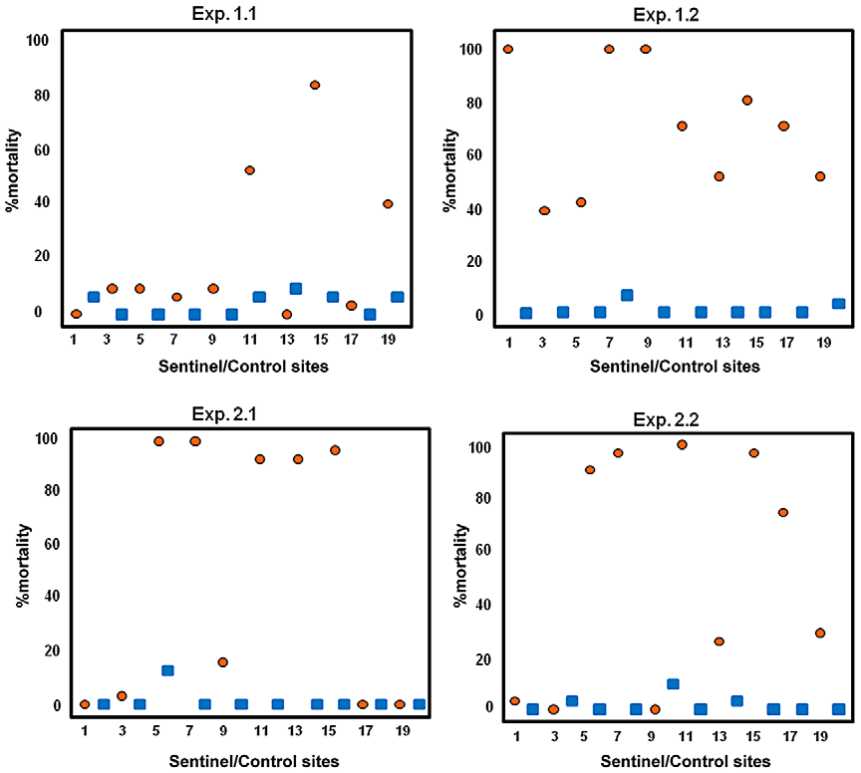
Overall mortality (%) in sentinel and control sites during the four experiments carried out in Rome. Experiment 1.1: 0,5% pyriproxyfen concentration; experiments 1.2, 2.1, 2.2: 5% pyriproxyfen concentration. Orange circles: sentinel sites numbered accordingly to Figures 2 and 3. Blue squares: control sites.

Figure 4 shows mortality in SS in the four experiments. In Exp 1.1 mortality was concentrated in 3 out of 10 SS, while in Exp 1.2 it ranged between 40 and 100%. In both Site 2 experiments mortality >76% was observed in 5 out of 10 SS, 4 of which were located in the same position in both replicates.

## Discussion

A number of experiments carried out under laboratory conditions have shown that adult *Ae. albopictus* topically contaminated with PPF can transfer enough material to water containers to exert a significant lethal effect on pre-imaginal stages developing therein [23], [20], [29], [30]. For the first time we tested the hypothesis that wild *Ae. albopictus* adults can act as “auto-disseminators” of PPF and inhibit adult emergence from sentinel sites. Although it was not possible to measure PPF concentration in those sites (the low doses of the product are beneath the limits of detection for any published method), the evidence supports the working hypothesis and suggests that auto-dissemination could represent a valid, novel approach for reducing *Ae. albopictus* densities in urban temperate areas.

We observed significantly higher mortality in our sentinel sites than in the controls. This shows that PPF was transferred by mosquitoes into sentinel sites and elicited a lethal effect. Mortality was almost exclusively limited to the pupal stage, i.e. the stage on which PPF is known to have its major effect [26]. Note that control sites were exposed to exactly the same experimental conditions as sentinel sites, except for the fact that contact with potentially contaminated mosquitoes was prevented by a net cover in the controls.

Mortality was not uniformly distributed among sentinel sites. This strongly suggests that PPF contamination occurred at some sites, but not others - presumably as a result of differences in the frequency of visits made by contaminated mosquitoes. This is particularly evident in Site 2, where mortality was observed in 5 out of 10 SSs, 4 of which were located at the same position in both replicates (Figures 3 and 4), leading to hypothesize that these sites were more attractive for the mosquitoes than the remaining ones. The same applies for the first replicate in Site 1. In the second replicate, however, mortality ranging from 40 to 100% was observed in all SSs suggesting that these were all visited and contaminated but with differing frequency.

In the cemetery experiments, a 10-fold increase in PPF concentration resulted in a 3-fold increase in mortality (Table 2).

Median mortality in sentinel-sites, in the three experiments carried out with a 5% PPF concentration, was higher in Site 1 than in Site 2 (18 and 13 deaths/25 larvae, respectively). In the mixed-effect logistic regression model, the variance due to the “site effect” was 0.12 (Standard deviation = 0.35) and 1.74 (Standard Deviation = 1.32), respectively. This is the likely consequence of the fact that, as mentioned above, in the Site 2 most mortality was observed only in 50% of the sentinel sites, while in Site 1 40–100% mortality was observed in every sentinel site. This presumably reflects the different ecology of the two experimental areas. Site 1, being an underground corridor, is ecologically very homogeneous and it is reasonable to hypothesize that all sentinel sites were equally attractive to the flying wild mosquitoes. The enclosed garden in Site 2 was far more heterogeneous and sentinel sites were located outdoors in a wider area compared to Site 1. It is reasonable to hypothesise that under these conditions, sentinel sites vary in attractiveness depending on contrast against background, shade, humidity, etc. However, even under such heterogeneous conditions the overall mortality in sentinel sites was about 37–49 fold higher than in controls. It is also important to emphasize that the experiments were not designed with the aim of identifying environmental factors that might optimise impact, although the effect of sun exposure has already been hypothesized in auto-dissemination experiments carried out against *Ae. aegypti* in Peru [21]. In fact, it is relevant to highlight that the major/minor attractiveness of sentinel sites to mosquitoes is likely to affect the results of the experiments, but has a relatively low practical relevance. In fact, if the approach is applied to reduce *Ae. albopictus* adult densities, the mosquitoes themselves will disseminate PPF to the most attractive (i.e. most productive) natural breeding sites. Further studies are required to assess the ideal conditions for the location of dissemination stations, as well as to evaluate the persistence of the lethal effect under different environmental (e.g. temperatures, sun exposures) and ecological (e.g. air flow, presence of animals, of humans, other disturbances/attractions) variables.

In the only other auto-dissemination experiment ever carried out in the field outdoors (i.e. in Peru with a 0.5% PPF concentration [21]), the overall reductions in *Ae. aegypti* adult emergence was 49–84%, as opposed to a 7–8% mortality in controls. In the single experiment carried out in Rome with the same PPF concentration, the overall reduction in *Ae. albopictus* adult emergence was 20.8%, as opposed to a 2.4% mortality in controls. This is quite encouraging: in fact a lower overall effect could have been expected in our experiments, since PPF LC_50_ reported for *Ae. albopictus* (0,11 ppb [25]) is about 10 times higher than that reported for *Ae. aegypti* (0.011 ppb [23]; 0.0039 ppb [24]). It is also worth noting that in Rome control sites were monitored concurrently to sentinel sites and were located at the same distance from dissemination stations, while in Peru they were separated in time. Thus, our results rule out the hypothesis that PPF could be passively transported by wind. If this had occurred, we would have expected higher mortality in at least some control sites.

Overall, the results from these small-scale experiments carried out in Rome strongly encourage further studies to evaluate the feasibility of the exploitation of the auto-dissemination approach to control *Ae. albopictus* densities in urban areas. In fact, the experimental design probably underestimates the overall impact of the approach, as other known effects of PPF on mosquitoes – such as sterilizing effects on adult females [28] and a decrease in male spermiogenesis [27] - were not taken into consideration. Moreover, the effect of auto-disseminated PPF was only monitored on third instar larvae (or later stages), while in natural breeding sites younger larvae will also be contaminated, presumably increasing the overall impact. The auto-dissemination approach has the potential to effectively counter the main challenge to conventional larviciding approaches by effectively targeting the myriad of cryptic breeding sites that these mosquitoes utilise. Based on results from mark-release-recapture experiments carried out in the campus of Sapienza University in Rome [33], it is possible to hypothesize that gravid, PPF-contaminated *Ae. albopictus* females could contaminate breeding sites in a 200-m radius area around a dissemination station. Other relevant advantages of the auto-dissemination approach are: i) a higher residual activity of PPF (4 months in water [34]) compared to that of other compounds commonly used for larval control and ii) no risk for human health, due to the low-toxicity of the product for vertebrates and the high sensitivity of mosquito larvae/pupae [35]. With regard to this latter point, although PPF is effective against many insects, the proposed approach targets container-breeding species with such tiny amounts of compound, disseminated exclusively to their breeding sites, that impacts on non-target species are likely to be minimal. Finally, the auto-dissemination approach could be a very cost-effective control tool. Once deployed, the dissemination station does not require frequent maintenance, nor frequent toxicant applications, thanks to PPF's outstanding persistence [28]. It is also of note that other Culicidae species (e.g. *Culex pipiens*, a common nuisance species in urban Italy, sharing some *Ae. albopictus* breeding sites) may contribute to the dissemination of the product [36] and be affected by it themselves. Indeed, larger-scale field experiments are needed to evaluate more precisely the worldwide feasibility of the approach and to promote its use against *Ae. albopictus*.

With appropriate modifications to the dissemination stations, auto-dissemination tools may be simple enough to be deployed by members of the public. In the experiments carried out in Rome, PPF dissemination stations were adapted from existing sticky-traps for collecting *Ae. albopictus* and *Ae. aegypti* females [31], [37]. The sticky surfaces were replaced with cloth surfaces dusted with pulverized PPF. In Peru, dissemination stations consisted of 1-liter plastic disposable pots lined with damp black cloth, These dissemination tools could be optimized to increase their attractiveness for mosquitoes and their overall practicability. The fabric of the cloths could be optimized for the release of PPF to mosquito legs, and the method of application of the compound into the cloth could be optimized and standardized. Other methods to attract and contaminate adult mosquitoes could be developed (see, for instance, [30]). Finally, the auto-dissemination approach could be exploited to spread other mosquito toxic compounds, such as other juvenile hormones or fungi, which might have an even greater impact than PPF [38], [39], [40], [41].

## References

[1] Hawley WA (1988) The biology of Aedes albopictus. Journal of the American Mosquito Control Association Supplement 1: 1. 3068349

[2] Scholte EJ, Schaffner F (2007) 14. Waiting for the tiger: establishment and spread of the Aedes albopictus mosquito in Europe. Emerging pests and vector-borne diseases in Europe 1: 241.

[3] Renault P, Solet J-L, Sissoko D, Balleydier E, Larrieu S, et al (2007) A major epidemic of Chikungunya virus infection on Reunion Island, France, 2005–2006. American Journal of Tropical Medicine & Hygiene 77: 727–731. 17978079

[4] De Lamballerie X, Leroy E, Charrel RN, Ttsetsarkin K, Higgs S, et al (2008) Chikungunya virus adapts to tiger mosquito via evolutionary convergence: a sign of things to come. Virology Journal 5: 422X–5. 10.1186/1743-422X-5-33 PMC2266737 18304328

[5] Kumar NP, Joseph R, Kamaraj T, Jambulingam P (2008) A226V mutation in virus during the 2007 Chikungunya outbreak in Kerala, India. Journal of General Virology 89: 1945–1948 doi:10.1099/vir.0.83628-0. 18632966 10.1099/vir.0.83628-0

[6] Rezza G, Nicoletti L, Angelini R, Romi R, Finarelli AC, et al (2007) Infection with Chikungunya virus in Italy: an outbreak in a temperate region. Lancet 370: 1840–1846 doi:10.1016/S0140-6736(07)61779-6. 18061059 10.1016/S0140-6736(07)61779-6

[7] Beltrame A, Angheben A, Bisoffi Z, Monteiro G, Marocco S, et al (2007) Imported Chikungunya infection, Italy. Emerging Infectious Diseases 13: 1264. 17953112 10.3201/eid1308.070161 PMC2828087

[8] Calzolari M, Bonilauri P, Bellini R, Caimi M, Defilippo F, et al (2010) Arboviral survey of mosquitoes in two northern Italian regions in 2007 and 2008. Vector Borne & Zoonotic Diseseas 10: 875–884 doi:10.1089/vbz.2009.0176. 10.1089/vbz.2009.0176 20370434

[9] Calzolari M, Bonilauri P, Bellini R, Albieri A, Defilippo F, et al (2010) Evidence of Simultaneous Circulation of West Nile and Usutu Viruses in Mosquitoes Sampled in Emilia-Romagna Region (Italy) in 2009. PLoS ONE 5: e14324 doi:10.1371/journal.pone.0014324. 21179462 10.1371/journal.pone.0014324 PMC3002278

[10] La Ruche G, Souarès Y, Armengaud A, Peloux-Petiot F, Delaunay P, et al (2010) First two autochthonous Dengue virus infections in metropolitan France, September 2010. Euro Surveillance 15: 19676. 20929659

[11] Peng HJ, Lai HB, Zhang QL, Xu BY, Zhang H, et al (2012) A local outbreak of Dengue caused by an imported case in Dongguan China. BMC Public Health 12: 83. 22276682 10.1186/1471-2458-12-83 PMC3311058

[12] Valerio L, Marini F, Bongiorno G, Facchinelli L, Pombi M, et al (2010) Host-feeding patterns of Aedes albopictus (Diptera: Culicidae) in urban and rural contexts within Rome Province, Italy. Vector-Borne & Zoonotic Diseases 10: 291–294. 19485771 10.1089/vbz.2009.0007

[13] Sabatini A, Raineri V, Trovato G, Coluzzi M (1990) others (1990) [Aedes albopictus in Italy and possible diffusion of the species into the Mediterranean area]. Parassitologia 32: 301. 2132441

[14] Dalla Pozza G, Majori G (1992) First record of Aedes albopictus establishment in Italy. Journal of the American Mosquito Control Association 8: 318–320. 1402871

[15] Romi R, Majori G (2008) An overview of the lesson learned in almost 20 years of fight against the “tiger” mosquito. Parassitologia 50: 117–119. 18693574

[16] Romi R, Di Luca M, Majori G (1999) Current status of Aedes albopictus and Aedes atropalpus in Italy. Journal of the American Mosquito Control Association 15: 425–427. 10480136

[17] Toma L, Severini F, Di Luca M, Bella A, Romi R (2003) Seasonal patterns of oviposition and egg hatching rate of Aedes albopictus in Rome. Journal of the American Mosquito Control Association 19: 19–22. 12674530

[18] Pombi M, Costantini C, della Torre A (2003) [Aedes albopictus (Diptera: Culicidae) in Rome: experimental study of relevant control strategy parameters]. Parassitologia 45: 97–102. 15267004

[19] Romi R, Toma L, Severini F, Luca M, Boccolini D, et al.. (2009) Guidelines for control of potential arbovirus mosquito vectors in Italy. Rapporti ISTISAN-Istituto Superiore di Sanità.

[20] Itoh T (1994) Utilization of Blood fed Females of Aedes aegypti as a Vehicle for the Transfer of the Insect Growth Regulator, Pyriproxyfen, to Larval Habitats. Tropical Medicine 36: 243–248. 7807075

[21] Devine GJ, Perea EZ, Killeen GF, Stancil JD, Clark SJ, et al (2009) Using adult mosquitoes to transfer insecticides to Aedes aegypti larval habitats. Proceedings of the National Academy of Science USA 106: 11530–11534 doi:10.1073/pnas.0901369106. 10.1073/pnas.0901369106 PMC2702255 19561295

[22] Trexler JD, Apperson CS, Schal C (1998) Laboratory and field evaluations of oviposition responses of Aedes albopictus and Aedes triseriatus (Diptera: Culicidae) to oak leaf infusions. Journal of Medical Entomology 35: 967–976. 9835688 10.1093/jmedent/35.6.967

[23] Itoh T, Kawada H, Abe A, Eshita Y, Rongsriyam Y, et al (1994) Utilization of bloodfed females of Aedes aegypti as a vehicle for the transfer of the insect growth regulator pyriproxyfen to larval habitats. Journal of the American Mosquito Control Association 10: 344–347. 7807075

[24] Henrick C (1995) Juvenoids. Agrochemicals from natural products 147–213.

[25] Ali A, Nayar JK, Xue RD (1995) Comparative toxicity of selected larvicides and insect growth regulators to a Florida laboratory population of Aedes albopictus. Journal of the American Mosquito Control Association 11: 72–76. 7616194

[26] Hirano M, Hatakoshi M, Kawada H, Takimoto Y (1998) Pyriproxyfen and other juvenile hormone analogues. Reviews in Toxicology 2: 357–394.

[27] Iwanaga K, Kanda T (1988) The Effects of a Juvenile Hormone Active Oxime Ether Compound on the Metamorphosis and Reproduction of an Anopheline Vector, Anopheles balabacensis (Diptera: Culicidae). Applied Entomology and Zoology 23: 186–193.

[28] Sihuincha M, Zamora-Perea E, Orellana-Rios W, Stancil JD, López-Sifuentes V, et al (2005) Potential use of pyriproxyfen for control of Aedes aegypti (Diptera: Culicidae) in Iquitos, Perú. Journal of Medical Entomology 42: 620–630. 16119551 10.1093/jmedent/42.4.620

[29] Dell Chism B, Apperson CS (2003) Horizontal transfer of the insect growth regulator pyriproxyfen to larval microcosms by gravid Aedes albopictus and Ochlerotatus triseriatus mosquitoes in the laboratory. Medical and Veterinary Entomology 17: 211–220. 12823839 10.1046/j.1365-2915.2003.00433.x

[30] Gaugler R, Suman D, Wang Y (2012) An autodissemination station for the transfer of an insect growth regulator to mosquito oviposition sites. Medical and Veterinary Entomology 26: 37–45 doi:10.1111/j.1365-2915.2011.00970.x. 21689125 10.1111/j.1365-2915.2011.00970.x

[31] Facchinelli L, Valerio L, Pombi M, Reiter P, Costantini C, et al (2007) Development of a novel sticky trap for container-breeding mosquitoes and evaluation of its sampling properties to monitor urban populations of Aedes albopictus. Medical and Veterinary Entomology 21: 183–195 doi:10.1111/j.1365-2915.2007.00680.x. 17550438 10.1111/j.1365-2915.2007.00680.x

[32] Bagheri F, Talebi K, Hosseininaveh V (2011) Biological effects of the juvenoid pyriproxyfen on the pistachio green stink bug, Brachynema germari Kol. (Hem.: Pentatomidae). Archives ff Phytopathology and Plant Protection 44: 1273–1284 doi:10.1080/03235408.2010.493749.

[33] Marini F, Caputo B, Pombi M, Tarsitani G, della Torre A (2010) Study of Aedes albopictus dispersal in Rome, Italy, using sticky traps in mark-release-recapture experiments. Medical and Veterinary Entomology 24: 361–368 doi:10.1111/j.1365-2915.2010.00898.x. 20666995 10.1111/j.1365-2915.2010.00898.x

[34] Seccacini E, Lucia A, Harburguer L, Zerba E, Licastro S, et al (2008) Effectiveness of pyriproxyfen and diflubenzuron formulations as larvicides against Aedes aegypti. Journal of American Mosquito Control Association 24: 398–403. 10.2987/5697.1 18939692

[35] World Health Organization (2007) Pyriproxyfen in Drinking-water: Use for Vector Control in Drinking-water Sources and Containers.

[36] Devine GJ, Killeen JF (2010) The potential of a new larviciding method for the control of malaria vectors. Malaria Journal 9: 142. 20500865 10.1186/1475-2875-9-142 PMC2895607

[37] Facchinelli L, Koenraadt CJM, Fanello C, Kijchalao U, Valerio L, et al (2008) Evaluation of a Sticky Trap for Collecting Aedes (Stegomyia) Adults in a Dengue-Endemic Area in Thailand. American Journal of Tropical Medicine & Hygiene 78: 904–909. 18541767

[38] Becnel JJ, Johnson MA (2000) Impact of Edhazardia aedis (Microsporidia: Culicosporidae) on a Seminatural Population of Aedes aegypti (Diptera: Culicidae). Biological Control 18: 39–48 doi:10.1006/bcon.1999.0805.

[39] Scholte E-J, Knols BG, Takken W (2004) Autodissemination of the entomopathogenic fungus Metarhizium anisopliae amongst adults of the malaria vector Anopheles gambiae s.s. Malaria Journal 3: 45 doi:10.1186/1475-2875-3-45. 15566626 10.1186/1475-2875-3-45 PMC535890

[40] Reyes-Villanueva F, Garza-Hernandez JA, Garcia-Munguia AM, Tamez-Guerra P, Howard AF, Rodriguez-Perez MA (2011) Dissemination of Metarhizium anisopliae of low and high virulence by mating behavior in Aedes aegypti. Parasite & Vectors 4: 171 doi:10.1186/1756-3305-4-171. 10.1186/1756-3305-4-171 PMC3178524 21906283

[41] García-Munguía AM, Garza-Hernández JA, Rebollar-Tellez EA, Rodríguez-Pérez MA, Reyes-Villanueva F (2011) Transmission of Beauveria bassiana from male to female Aedes aegypti mosquitoes. Parasite & Vectors 4: 24 doi:10.1186/1756-3305-4-24. 10.1186/1756-3305-4-24 PMC3051917 21352560

